# Crystal structure of flumioxazin

**DOI:** 10.1107/S2056989015017223

**Published:** 2015-09-17

**Authors:** Hyunjin Park, Jineun Kim, Eunjin Kwon, Tae Ho Kim

**Affiliations:** a Department of Chemistry and Research Institute of Natural Sciences, Gyeongsang National University, Jinju 52828, Republic of Korea

**Keywords:** crystal structure, dicarboximide herbicide, flumioxazin, 1*H*-iso­indole, 1,4-benzoxazine, C—H⋯F hydrogen bonds

## Abstract

The title compound {systematic name: 2-[7-fluoro-3,4-di­hydro-3-oxo-4-(prop-2-yn-1-yl)-2*H*-1,4-benzoxazin-6-yl]-4,5,6,7-tetra­hydro-1*H*-iso­indole-1,3(2*H*)-dione}, C_19_H_15_FN_2_O_4_, is a dicarboximide herbicide. The dihedral angle between the male­imide and benzene ring planes is 66.13 (5)°. In the crystal, C—H⋯O and C—H⋯F hydrogen bonds and weak C—H⋯π inter­actions [3.5601 (19) Å] link adjacent mol­ecules, forming two-dimensional networks extending parallel to the (110) plane.

## Related literature   

For information on the herbicidal properties of the title compound, see: Saladin *et al.* (2003 ▸); Geoffroy *et al.* (2004 ▸). For a related crystal structure, see: Hou *et al.* (2004 ▸).
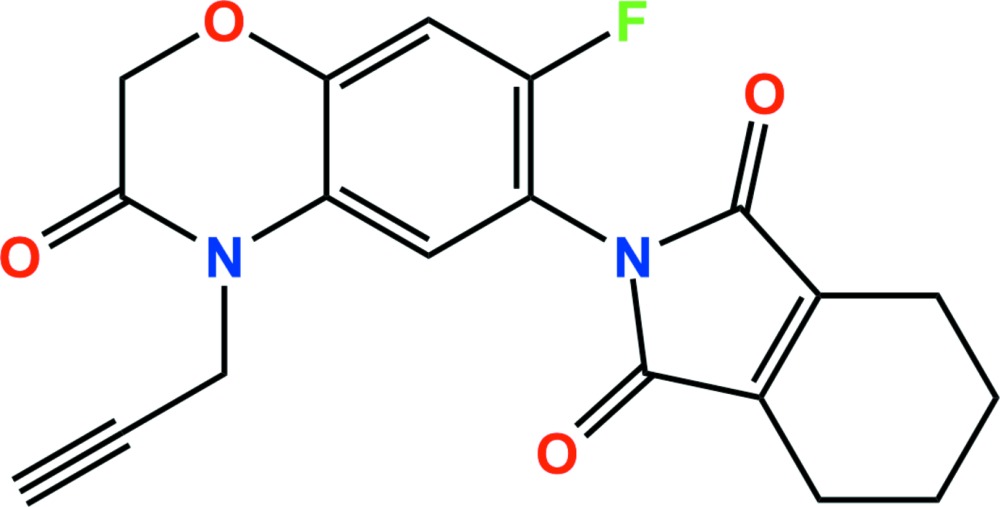


## Experimental   

### Crystal data  

C_19_H_15_FN_2_O_4_
*M* *_r_* = 354.33
Monoclinic, 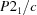

*a* = 8.896 (1) Å
*b* = 7.1592 (8) Å
*c* = 25.708 (3) Å
β = 96.039 (6)°
*V* = 1628.2 (3) Å^3^
*Z* = 4
Mo *K*α radiation
μ = 0.11 mm^−1^
*T* = 173 K
0.50 × 0.26 × 0.05 mm

### Data collection  

Bruker APEXII CCD diffractometer
Absorption correction: multi-scan (*SADABS*; Bruker, 2013 ▸) *T*_min_ = 0.575, *T*_max_ = 0.746
27372 measured reflections
4067 independent reflections
3277 reflections with *I* > 2σ(*I*)
*R*_int_ = 0.067

### Refinement  

*R*[*F* ^2^ > 2σ(*F* ^2^)] = 0.052
*wR*(*F* ^2^) = 0.137
*S* = 1.07
4067 reflections
235 parameters
H-atom parameters constrained
Δρ_max_ = 0.32 e Å^−3^
Δρ_min_ = −0.27 e Å^−3^

### 

Data collection: *APEX2* (Bruker, 2013 ▸); cell refinement: *SAINT* (Bruker, 2013 ▸); data reduction: *SAINT*; program(s) used to solve structure: *SHELXS97* (Sheldrick, 2008 ▸); program(s) used to refine structure: *SHELXL2013* (Sheldrick, 2015 ▸); molecular graphics: *DIAMOND* (Brandenburg, 2010 ▸); software used to prepare material for publication: *SHELXTL* (Sheldrick, 2008 ▸).

## Supplementary Material

Crystal structure: contains datablock(s) global, I. DOI: 10.1107/S2056989015017223/hb7502sup1.cif

Structure factors: contains datablock(s) I. DOI: 10.1107/S2056989015017223/hb7502Isup2.hkl

Supporting information file. DOI: 10.1107/S2056989015017223/hb7502Isup3.cml

**Fig. 1.**
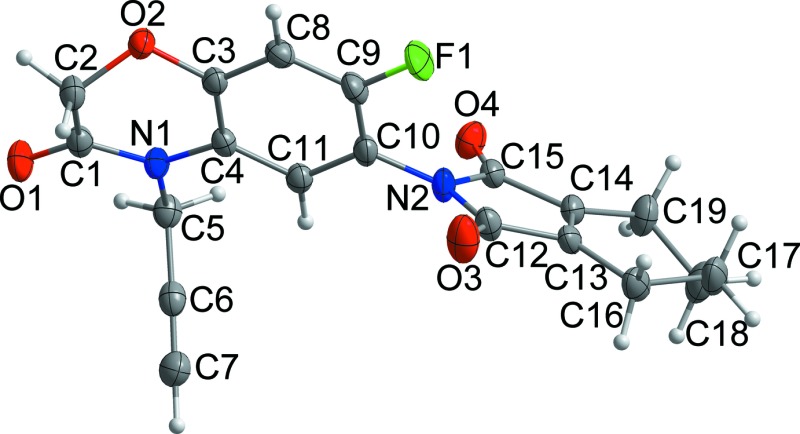
The asymmetric unit of the title compound with the atom-numbering scheme. Displacement ellipsoids are drawn at the 50% probability level. H atoms are shown as small spheres of arbitrary radius.

**Fig. 2.**
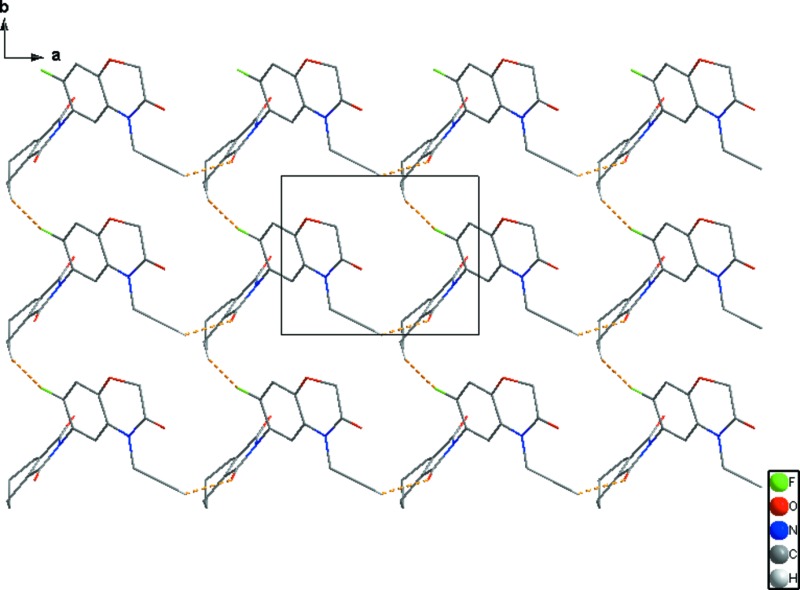
Crystal packing viewed along the c axis. The intermolecular interactions are shown as dashed lines.

. DOI: 10.1107/S2056989015017223/hb7502fig1.tif
The asymmetric unit of the title compound with the atom-numbering scheme. Displacement ellipsoids are drawn at the 50% probability level. H atoms are shown as small spheres of arbitrary radius.

c . DOI: 10.1107/S2056989015017223/hb7502fig2.tif
Crystal packing viewed along the *c* axis. The inter­molecular inter­actions are shown as dashed lines.

CCDC reference: 1424397

Additional supporting information:  crystallographic information; 3D view; checkCIF report

## Figures and Tables

**Table 1:** Hydrogen-bond geometry (, ) *Cg*1 is the centroid of the C3/C4/C8C11 ring.

*D*H*A*	*D*H	H*A*	*D* *A*	*D*H*A*
C7H7O4^i^	0.95	2.39	3.178(2)	140
C19H19*B*F1^ii^	0.99	2.36	3.289(2)	155
C16H16*A* *Cg*1^iii^	0.99	2.63	3.5601(19)	157

Symmetry codes: (i) 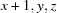; (ii) 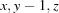; (iii) 
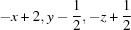
.

## supplementary crystallographic information

### S1. Comment

Flumioxazin [systematic name: 2-[7-fluoro-3,4-dihydro-3-oxo-4-(2-propyn-1-yl)-2*H*-1,4-benzoxazin-6-yl]-4,5,6,7-tetrahydro-1*H*-isoindole-1,3(2*H*)-dione] is a soil applied pre-emergent herbicide used to inhibit development of redroot pigweed. (Geoffroy *et al.*, 2004; Saladin *et al.*, 2003). However, its crystal structure has not been reported until now. In the title compound (Fig. 1), the dihedral angle between pyrrole and benzne rings is 66.13 (5)°. All bond lengths and bond angles are normal and comparable to those observed in a similar crystal structure (Hou *et al.*, 2004).

In the crystal structure (Fig. 2), C—H···O and C—H···F hydrogen bonds and weak C–H···π interactions are observed (Table 1). Two-dimensional networks are formed by the hydrogen bonds and weak C–H···π interactions.

### S2. Experimental

The title compound was purchased from the Dr Ehrenstorfer GmbH Company. Slow evaporation of a solution in CH_3_CN gave single crystals suitable for X-ray analysis.

### S3. Refinement

All H-atoms were positioned geometrically and refined using a riding model with d(C—H) = 0.99 Å, *U*_iso_ = 1.2*U*_eq_(C) for CH_2_ group, d(C—H) = 0.95 Å, *U*_iso_ = 1.2*U*_eq_(C) for aromatic C—H aromatic C—H and C*sp*—H.

### Crystal data

**Table:**

C_19_H_15_FN_2_O_4_	*F*(000) = 736
*M**_r_* = 354.33	*D*_x_ = 1.445 Mg m^−^^3^
Monoclinic, *P*2_1_/*c*	Mo *K*α radiation, λ = 0.71073 Å
*a* = 8.896 (1) Å	Cell parameters from 8497 reflections
*b* = 7.1592 (8) Å	θ = 2.7–28.2°
*c* = 25.708 (3) Å	µ = 0.11 mm^−^^1^
β = 96.039 (6)°	*T* = 173 K
*V* = 1628.2 (3) Å^3^	Plate, colourless
*Z* = 4	0.50 × 0.26 × 0.05 mm

### Data collection

**Table:**

Bruker APEXII CCD diffractometer	3277 reflections with *I* > 2σ(*I*)
φ and ω scans	*R*_int_ = 0.067
Absorption correction: multi-scan (*SADABS*; Bruker, 2013)	θ_max_ = 28.4°, θ_min_ = 1.6°
*T*_min_ = 0.575, *T*_max_ = 0.746	*h* = −11→11
27372 measured reflections	*k* = −9→9
4067 independent reflections	*l* = −34→34

### Refinement

**Table:**

Refinement on *F*^2^	0 restraints
Least-squares matrix: full	Hydrogen site location: inferred from neighbouring sites
*R*[*F*^2^ > 2σ(*F*^2^)] = 0.052	H-atom parameters constrained
*wR*(*F*^2^) = 0.137	*w* = 1/[σ^2^(*F*_o_^2^) + (0.0535*P*)^2^ + 0.9028*P*] where *P* = (*F*_o_^2^ + 2*F*_c_^2^)/3
*S* = 1.07	(Δ/σ)_max_ < 0.001
4067 reflections	Δρ_max_ = 0.32 e Å^−^^3^
235 parameters	Δρ_min_ = −0.27 e Å^−^^3^

### Special details

**Table:**

Geometry. All e.s.d.'s (except the e.s.d. in the dihedral angle between two l.s. planes) are estimated using the full covariance matrix. The cell e.s.d.'s are taken into account individually in the estimation of e.s.d.'s in distances, angles and torsion angles; correlations between e.s.d.'s in cell parameters are only used when they are defined by crystal symmetry. An approximate (isotropic) treatment of cell e.s.d.'s is used for estimating e.s.d.'s involving l.s. planes.

### Fractional atomic coordinates and isotropic or equivalent isotropic displacement parameters (Å^2^)

**Table:**

	*x*	*y*	*z*	*U*_iso_*/*U*_eq_	
F1	0.77768 (12)	0.66568 (18)	0.33755 (5)	0.0431 (3)	
O1	1.40190 (15)	0.41585 (19)	0.54636 (5)	0.0359 (3)	
O2	1.12467 (13)	0.73087 (17)	0.48786 (4)	0.0273 (3)	
O3	0.94166 (16)	0.4892 (2)	0.25135 (5)	0.0459 (4)	
O4	0.74322 (15)	0.08897 (18)	0.36415 (5)	0.0331 (3)	
N1	1.22083 (15)	0.3652 (2)	0.47885 (5)	0.0235 (3)	
N2	0.86942 (16)	0.3094 (2)	0.31951 (5)	0.0276 (3)	
C1	1.30559 (18)	0.4767 (3)	0.51371 (6)	0.0265 (4)	
C2	1.27631 (19)	0.6824 (3)	0.50845 (7)	0.0291 (4)	
H2A	1.2983	0.7410	0.5433	0.035*	
H2B	1.3474	0.7360	0.4853	0.035*	
C3	1.06753 (17)	0.6259 (2)	0.44577 (6)	0.0226 (3)	
C4	1.11458 (17)	0.4423 (2)	0.43988 (6)	0.0217 (3)	
C5	1.2461 (2)	0.1636 (2)	0.48166 (6)	0.0272 (4)	
H5A	1.1481	0.0987	0.4739	0.033*	
H5B	1.2872	0.1300	0.5177	0.033*	
C6	1.3508 (2)	0.0983 (2)	0.44508 (7)	0.0284 (4)	
C7	1.4371 (2)	0.0447 (3)	0.41648 (7)	0.0357 (4)	
H7	1.5064	0.0015	0.3935	0.043*	
C8	0.95463 (18)	0.7036 (3)	0.41121 (6)	0.0266 (4)	
H8	0.9225	0.8288	0.4154	0.032*	
C9	0.89012 (18)	0.5945 (3)	0.37066 (7)	0.0284 (4)	
C10	0.93760 (18)	0.4142 (3)	0.36273 (6)	0.0261 (4)	
C11	1.05195 (18)	0.3384 (2)	0.39736 (6)	0.0244 (3)	
H11	1.0873	0.2154	0.3920	0.029*	
C12	0.87139 (19)	0.3604 (3)	0.26659 (7)	0.0298 (4)	
C13	0.76914 (17)	0.2264 (3)	0.23609 (6)	0.0252 (4)	
C14	0.70932 (18)	0.1121 (2)	0.26880 (6)	0.0246 (4)	
C15	0.77039 (17)	0.1593 (2)	0.32346 (6)	0.0235 (3)	
C16	0.7429 (2)	0.2189 (3)	0.17802 (6)	0.0338 (4)	
H16A	0.8355	0.1746	0.1637	0.041*	
H16B	0.7193	0.3457	0.1640	0.041*	
C17	0.6125 (2)	0.0878 (3)	0.16111 (7)	0.0430 (5)	
H17A	0.5158	0.1542	0.1634	0.052*	
H17B	0.6171	0.0519	0.1241	0.052*	
C18	0.6158 (3)	−0.0865 (3)	0.19432 (8)	0.0468 (5)	
H18A	0.7113	−0.1546	0.1912	0.056*	
H18B	0.5311	−0.1690	0.1809	0.056*	
C19	0.6033 (2)	−0.0436 (3)	0.25236 (7)	0.0361 (4)	
H19A	0.4984	−0.0070	0.2573	0.043*	
H19B	0.6297	−0.1559	0.2739	0.043*	

### Atomic displacement parameters (Å^2^)

**Table:**

	*U*^11^	*U*^22^	*U*^33^	*U*^12^	*U*^13^	*U*^23^
F1	0.0319 (6)	0.0490 (7)	0.0443 (6)	0.0126 (5)	−0.0160 (5)	−0.0011 (5)
O1	0.0353 (7)	0.0380 (8)	0.0310 (6)	−0.0010 (6)	−0.0128 (5)	0.0025 (6)
O2	0.0270 (6)	0.0264 (6)	0.0273 (6)	−0.0010 (5)	−0.0021 (5)	−0.0052 (5)
O3	0.0433 (8)	0.0572 (10)	0.0362 (7)	−0.0243 (7)	−0.0003 (6)	0.0082 (7)
O4	0.0408 (7)	0.0352 (7)	0.0228 (6)	−0.0063 (6)	0.0011 (5)	0.0015 (5)
N1	0.0241 (7)	0.0220 (7)	0.0230 (6)	0.0000 (6)	−0.0034 (5)	0.0015 (5)
N2	0.0235 (7)	0.0368 (8)	0.0213 (6)	−0.0068 (6)	−0.0036 (5)	−0.0004 (6)
C1	0.0234 (8)	0.0334 (9)	0.0222 (7)	−0.0031 (7)	−0.0004 (6)	0.0009 (7)
C2	0.0264 (8)	0.0290 (9)	0.0301 (8)	−0.0042 (7)	−0.0055 (7)	−0.0026 (7)
C3	0.0197 (7)	0.0258 (8)	0.0222 (7)	−0.0031 (6)	0.0012 (6)	−0.0014 (6)
C4	0.0174 (7)	0.0247 (8)	0.0224 (7)	−0.0019 (6)	0.0001 (6)	0.0022 (6)
C5	0.0296 (8)	0.0247 (9)	0.0263 (8)	−0.0005 (7)	−0.0016 (6)	0.0027 (7)
C6	0.0288 (8)	0.0234 (9)	0.0309 (8)	−0.0005 (7)	−0.0065 (7)	0.0005 (7)
C7	0.0341 (10)	0.0369 (11)	0.0354 (9)	−0.0024 (8)	0.0004 (8)	−0.0058 (8)
C8	0.0221 (8)	0.0269 (9)	0.0306 (8)	0.0032 (7)	0.0020 (6)	0.0001 (7)
C9	0.0196 (8)	0.0354 (10)	0.0289 (8)	0.0028 (7)	−0.0041 (6)	0.0036 (7)
C10	0.0208 (8)	0.0332 (9)	0.0235 (7)	−0.0050 (7)	−0.0018 (6)	−0.0023 (7)
C11	0.0220 (8)	0.0263 (8)	0.0245 (7)	−0.0026 (7)	0.0007 (6)	−0.0008 (7)
C12	0.0221 (8)	0.0410 (10)	0.0255 (8)	−0.0046 (8)	−0.0010 (6)	0.0024 (7)
C13	0.0176 (7)	0.0355 (9)	0.0217 (7)	0.0024 (7)	−0.0022 (6)	−0.0006 (7)
C14	0.0204 (7)	0.0300 (9)	0.0221 (7)	0.0014 (7)	−0.0031 (6)	−0.0020 (6)
C15	0.0192 (7)	0.0280 (8)	0.0226 (7)	0.0017 (6)	−0.0013 (6)	−0.0010 (6)
C16	0.0285 (9)	0.0515 (12)	0.0207 (8)	0.0011 (8)	−0.0011 (6)	0.0025 (8)
C17	0.0322 (10)	0.0718 (15)	0.0234 (8)	−0.0059 (10)	−0.0039 (7)	−0.0085 (9)
C18	0.0483 (12)	0.0536 (14)	0.0368 (10)	−0.0122 (10)	−0.0032 (9)	−0.0155 (10)
C19	0.0397 (10)	0.0367 (11)	0.0301 (9)	−0.0123 (9)	−0.0047 (7)	−0.0025 (8)

### Geometric parameters (Å, º)

**Table:**

F1—C9	1.3436 (19)	C7—H7	0.9500
O1—C1	1.215 (2)	C8—C9	1.378 (2)
O2—C3	1.3707 (19)	C8—H8	0.9500
O2—C2	1.439 (2)	C9—C10	1.380 (3)
O3—C12	1.203 (2)	C10—C11	1.390 (2)
O4—C15	1.208 (2)	C11—H11	0.9500
N1—C1	1.366 (2)	C12—C13	1.487 (2)
N1—C4	1.4153 (19)	C13—C14	1.325 (2)
N1—C5	1.461 (2)	C13—C16	1.488 (2)
N2—C15	1.400 (2)	C14—C15	1.491 (2)
N2—C12	1.410 (2)	C14—C19	1.493 (2)
N2—C10	1.422 (2)	C16—C17	1.520 (3)
C1—C2	1.499 (3)	C16—H16A	0.9900
C2—H2A	0.9900	C16—H16B	0.9900
C2—H2B	0.9900	C17—C18	1.511 (3)
C3—C8	1.386 (2)	C17—H17A	0.9900
C3—C4	1.392 (2)	C17—H17B	0.9900
C4—C11	1.390 (2)	C18—C19	1.539 (3)
C5—C6	1.468 (2)	C18—H18A	0.9900
C5—H5A	0.9900	C18—H18B	0.9900
C5—H5B	0.9900	C19—H19A	0.9900
C6—C7	1.182 (3)	C19—H19B	0.9900
			
C3—O2—C2	114.43 (13)	C4—C11—C10	120.04 (16)
C1—N1—C4	121.20 (14)	C4—C11—H11	120.0
C1—N1—C5	118.24 (14)	C10—C11—H11	120.0
C4—N1—C5	120.54 (13)	O3—C12—N2	124.88 (16)
C15—N2—C12	109.88 (13)	O3—C12—C13	129.26 (16)
C15—N2—C10	124.61 (14)	N2—C12—C13	105.84 (14)
C12—N2—C10	124.73 (15)	C14—C13—C12	109.15 (14)
O1—C1—N1	122.97 (17)	C14—C13—C16	125.71 (16)
O1—C1—C2	121.16 (16)	C12—C13—C16	125.12 (16)
N1—C1—C2	115.85 (14)	C13—C14—C15	109.11 (15)
O2—C2—C1	114.67 (14)	C13—C14—C19	124.47 (15)
O2—C2—H2A	108.6	C15—C14—C19	126.37 (15)
C1—C2—H2A	108.6	O4—C15—N2	124.57 (15)
O2—C2—H2B	108.6	O4—C15—C14	129.44 (16)
C1—C2—H2B	108.6	N2—C15—C14	105.98 (13)
H2A—C2—H2B	107.6	C13—C16—C17	110.12 (15)
O2—C3—C8	117.99 (15)	C13—C16—H16A	109.6
O2—C3—C4	120.85 (14)	C17—C16—H16A	109.6
C8—C3—C4	121.02 (15)	C13—C16—H16B	109.6
C11—C4—C3	119.39 (14)	C17—C16—H16B	109.6
C11—C4—N1	122.06 (15)	H16A—C16—H16B	108.2
C3—C4—N1	118.50 (14)	C18—C17—C16	112.33 (16)
N1—C5—C6	112.80 (14)	C18—C17—H17A	109.1
N1—C5—H5A	109.0	C16—C17—H17A	109.1
C6—C5—H5A	109.0	C18—C17—H17B	109.1
N1—C5—H5B	109.0	C16—C17—H17B	109.1
C6—C5—H5B	109.0	H17A—C17—H17B	107.9
H5A—C5—H5B	107.8	C17—C18—C19	112.57 (18)
C7—C6—C5	178.63 (19)	C17—C18—H18A	109.1
C6—C7—H7	180.0	C19—C18—H18A	109.1
C9—C8—C3	118.25 (16)	C17—C18—H18B	109.1
C9—C8—H8	120.9	C19—C18—H18B	109.1
C3—C8—H8	120.9	H18A—C18—H18B	107.8
F1—C9—C8	119.17 (16)	C14—C19—C18	108.39 (16)
F1—C9—C10	118.68 (15)	C14—C19—H19A	110.0
C8—C9—C10	122.15 (15)	C18—C19—H19A	110.0
C9—C10—C11	119.04 (15)	C14—C19—H19B	110.0
C9—C10—N2	119.79 (15)	C18—C19—H19B	110.0
C11—C10—N2	121.17 (16)	H19A—C19—H19B	108.4
			
C4—N1—C1—O1	−177.07 (15)	C3—C4—C11—C10	−3.5 (2)
C5—N1—C1—O1	0.9 (2)	N1—C4—C11—C10	173.89 (14)
C4—N1—C1—C2	1.1 (2)	C9—C10—C11—C4	1.4 (2)
C5—N1—C1—C2	179.13 (15)	N2—C10—C11—C4	−178.70 (15)
C3—O2—C2—C1	−44.1 (2)	C15—N2—C12—O3	177.05 (18)
O1—C1—C2—O2	−152.56 (16)	C10—N2—C12—O3	6.8 (3)
N1—C1—C2—O2	29.2 (2)	C15—N2—C12—C13	−1.89 (19)
C2—O2—C3—C8	−154.71 (15)	C10—N2—C12—C13	−172.15 (15)
C2—O2—C3—C4	29.4 (2)	O3—C12—C13—C14	−176.9 (2)
O2—C3—C4—C11	178.59 (14)	N2—C12—C13—C14	1.96 (19)
C8—C3—C4—C11	2.9 (2)	O3—C12—C13—C16	5.0 (3)
O2—C3—C4—N1	1.1 (2)	N2—C12—C13—C16	−176.16 (16)
C8—C3—C4—N1	−174.58 (14)	C12—C13—C14—C15	−1.26 (19)
C1—N1—C4—C11	165.50 (15)	C16—C13—C14—C15	176.84 (16)
C5—N1—C4—C11	−12.5 (2)	C12—C13—C14—C19	−178.74 (16)
C1—N1—C4—C3	−17.1 (2)	C16—C13—C14—C19	−0.6 (3)
C5—N1—C4—C3	164.92 (15)	C12—N2—C15—O4	−178.20 (17)
C1—N1—C5—C6	−95.61 (17)	C10—N2—C15—O4	−7.9 (3)
C4—N1—C5—C6	82.40 (19)	C12—N2—C15—C14	1.17 (18)
O2—C3—C8—C9	−176.00 (15)	C10—N2—C15—C14	171.45 (15)
C4—C3—C8—C9	−0.2 (2)	C13—C14—C15—O4	179.43 (18)
C3—C8—C9—F1	178.24 (15)	C19—C14—C15—O4	−3.2 (3)
C3—C8—C9—C10	−2.0 (3)	C13—C14—C15—N2	0.10 (19)
F1—C9—C10—C11	−178.83 (15)	C19—C14—C15—N2	177.52 (16)
C8—C9—C10—C11	1.4 (3)	C14—C13—C16—C17	11.8 (3)
F1—C9—C10—N2	1.2 (2)	C12—C13—C16—C17	−170.41 (17)
C8—C9—C10—N2	−178.51 (16)	C13—C16—C17—C18	−40.6 (2)
C15—N2—C10—C9	−108.0 (2)	C16—C17—C18—C19	60.9 (2)
C12—N2—C10—C9	60.9 (2)	C13—C14—C19—C18	17.6 (3)
C15—N2—C10—C11	72.1 (2)	C15—C14—C19—C18	−159.46 (17)
C12—N2—C10—C11	−119.05 (19)	C17—C18—C19—C14	−46.4 (2)

### Hydrogen-bond geometry (Å, º)

Cg1 is the centroid of the C3/C4/C8–C11 ring.

**Table:**

*D*—H···*A*	*D*—H	H···*A*	*D*···*A*	*D*—H···*A*
C7—H7···O4^i^	0.95	2.39	3.178 (2)	140
C19—H19*B*···F1^ii^	0.99	2.36	3.289 (2)	155
C16—H16*A*···*Cg*1^iii^	0.99	2.63	3.5601 (19)	157

Symmetry codes: (i) *x*+1, *y*, *z*; (ii) *x*, *y*−1, *z*; (iii) −*x*+2, *y*−1/2, −*z*+1/2.
