# Supplementary figures and images for: Crystal structure of flumioxazin
Source: Acta Crystallogr E Crystallogr Commun. 2015 Sep 17;71(Pt 10):o768. doi: 10.1107/S2056989015017223 (PMC4647385; doi:10.1107/S2056989015017223)

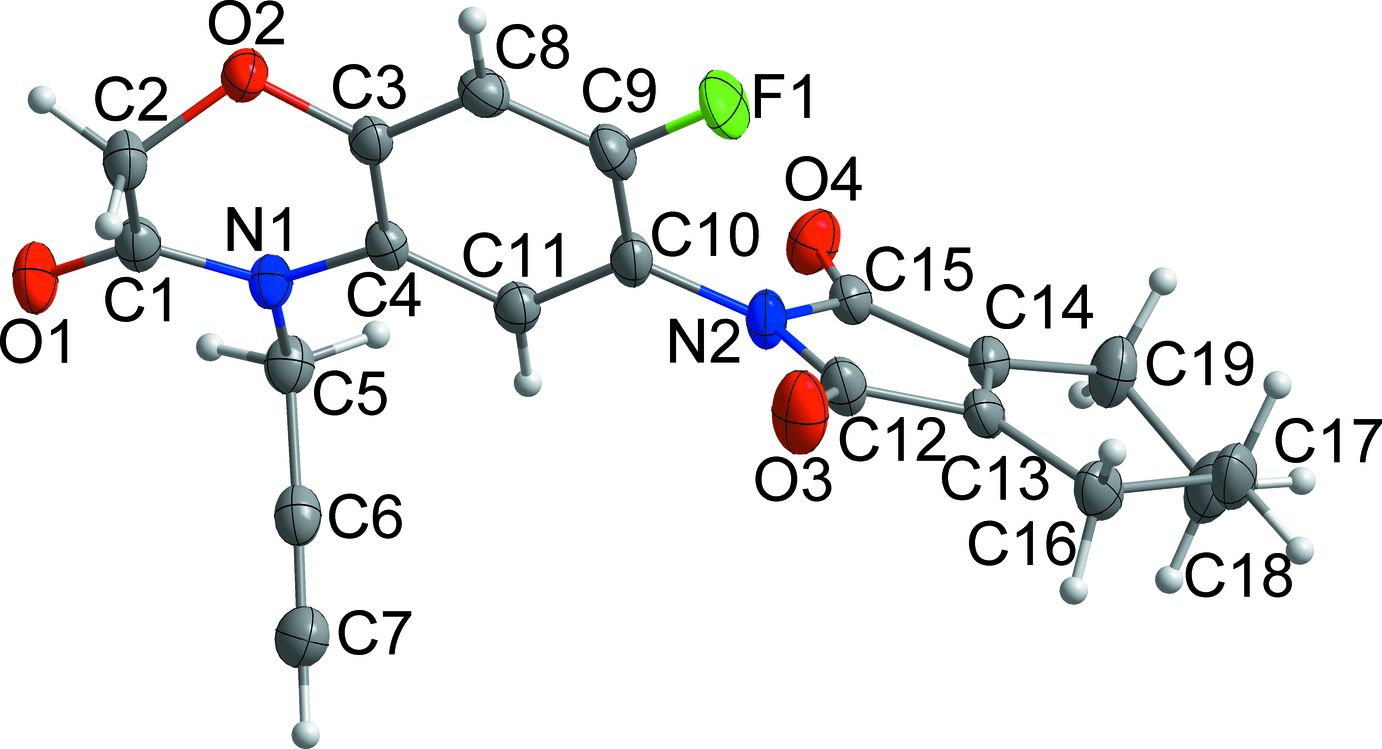

Supplement: Supplementary file 4 [file e-71-0o768-fig1.tif]

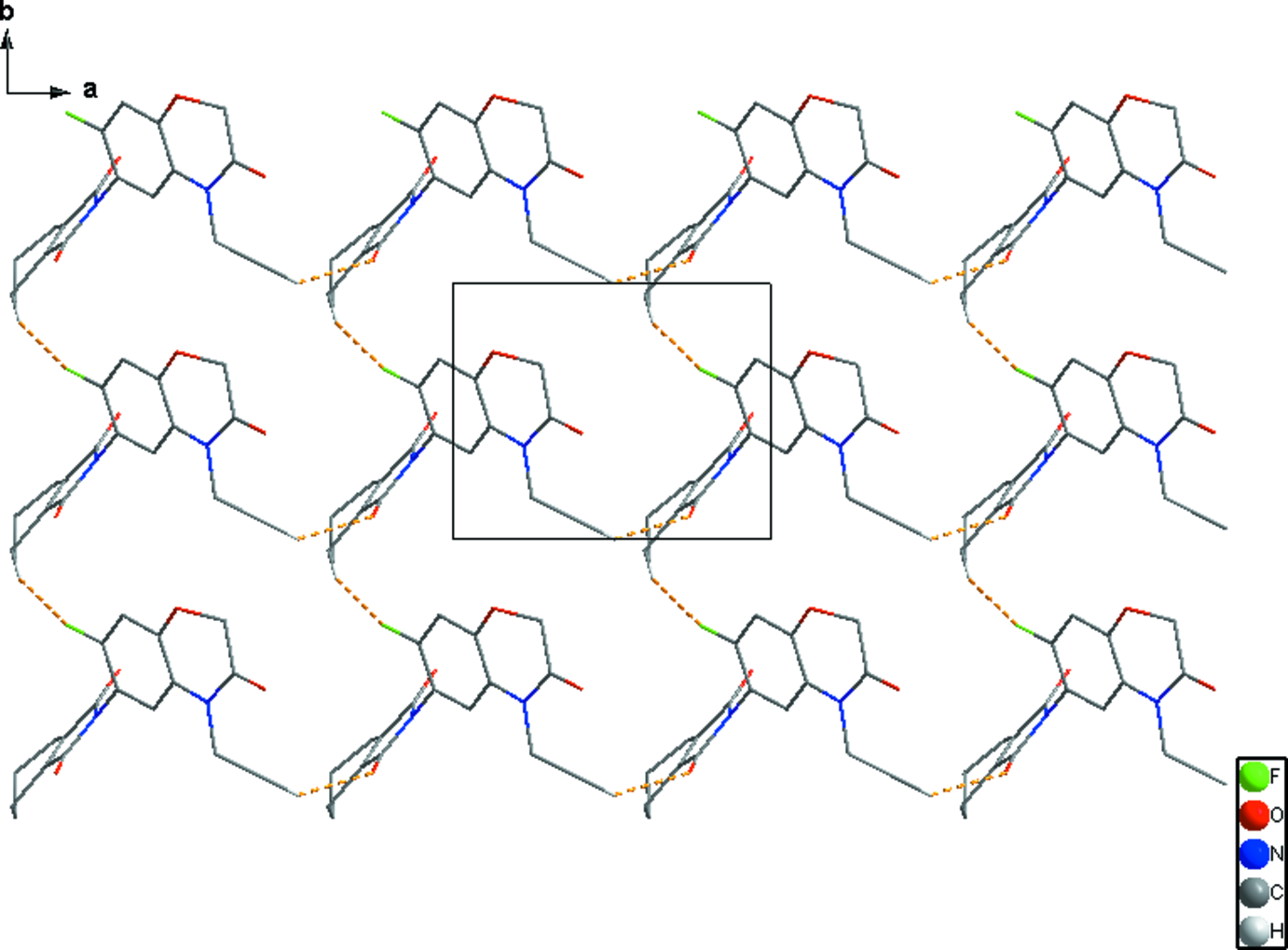

Supplement: Supplementary file 5 [file e-71-0o768-fig2.tif]
